# Positive affect is inversely related to the salience and emotion network’s connectivity

**DOI:** 10.1007/s11682-020-00397-1

**Published:** 2020-10-08

**Authors:** Di Qi, Charlene L. M. Lam, Jing Jun Wong, Dorita H. F. Chang, Tatia M. C. Lee

**Affiliations:** 1 State Key Laboratory of Brain and Cognitive Sciences, The University of Hong Kong, Hong Kong, China; 2 Laboratory of Neuropsychology and Human Neuroscience, The University of Hong Kong, Hong Kong, China; 3 Department of Psychology, The University of Hong Kong, Hong Kong, China

**Keywords:** Positive affect, Functional connectivity, Salience and emotion network, Dynamic causal modeling

## Abstract

Increasing evidence has shown that positive affect enhances many aspects of daily functioning. Yet, how dispositional positive affect is represented in the intrinsic brain networks remains unclear. Here, we used resting-state functional Magnetic Resonance Imaging to test how trait positive and negative affect of an individual were associated with the intrinsic connectivity of brain regions within the salience and emotion network and the default mode network in 70 healthy young adults. We observed that positive affect was negatively associated with connectivity within the salience and emotion network, particularly with the bidirectional connections spanning the left anterior insula and left nucleus accumbens. For connections between the salience and emotion network and the rest of the brain, we observed that positive affect was negatively related to the connectivity between the right amygdala and the right middle temporal gyrus. Affect-based modulations of connectivity were specific to positive affect and to the salience and emotion network. Our findings highlight the critical role of salience and emotion network in the neural relations of positive affect, and lay the groundwork for future studies on modeling the connectivity of salience and emotion network to predict mental well-being.

**Electronic supplementary material:**

The online version of this article (10.1007/s11682-020-00397-1) contains supplementary material, which is available to authorized users.

Positive affect (PA) refers to the manifestation of pleasant moods and emotions. It encompasses characteristics such as optimism, enthusiasm, confidence, and self-efficacy (Lyubomirsky et al. 2005). It is also associated with a number of beneficial health and social outcomes including improved mental and physical well-being (Davidson et al. 2010; Mauss et al. 2011). In view of the increasingly prevalent mood-related psychopathology, it is timely to understand the neural functional organization that underpins individual variation in the trait PA.

Structurally, PA was found to be positively correlated with hippocampal volume (Dennison et al. 2015). Functionally, individuals with a higher trait PA exhibited lower amygdala activation when viewing unpleasant stimuli (Sanchez et al. 2015). One study has directly investigated the brain’s functional organization of PA and negative affect (NA) using resting-state functional connectivity (FC). In this study, PA was found to be negatively associated with FC between dispersed brain areas across the whole brain, and the connectivity patterns of PA were dissociative from those of NA (Rohr et al. 2013). Their findings supported the potential for using resting-state functional magnetic resonance imaging (rs-fMRI) to investigate the affective organization in the brain, and prompted us to investigate the intrinsic brain networks relevant to PA, which could be further targeted during affect modulation.

The salience and emotion network (SEN), one of the most important intrinsic brain networks, is critical for integrating sensory, emotional, and cognitive information (Menon 2015). The SEN is a paralimbic-limbic network that contains both cortical and subcortical regions. The cortical regions include the anterior insula and the dorsal anterior cingulate cortex (dACC), while the subcortical structures include the amygdala, the ventral striatum, and the substantia nigra/ventral tegmental area. The cortical nodes are primarily responsible for detection and integration of salient sensory cues, action selection, and conflict monitoring, while the subcortical nuclei serve to integrate salient affective cues (Menon 2015). Dysfunction of the SEN has been observed in the psychopathology of several psychiatric disorders (Menon 2011). For instance, aberrant connectivity of anterior insula within the SEN was associated with the severity of symptoms in patients with major depressive disorder, of which lowered PA is one of the prominent characteristics (Manoliu et al. 2014). Furthermore, the ability to maintain positive was found to predict the connectivity strength between the amygdala and thalamostriatal regions in healthy adults (Rohr et al. 2015). Given SEN plays critical roles in affective experience (Seeley et al. 2007; Touroutoglou et al. 2014; Touroutoglou et al. 2012), and its connectivity was also found to be related to affective traits like trait drive (DelDonno et al. 2017), we hence speculate that SEN is also likely to be implicated in trait PA/NA.

In the current study, we sought to identify the intrinsic brain network connectivity underpinning affect using rs-fMRI. To this end, we averaged the FC between each node of the SEN to obtain a composite index of network connectivity called *SEN connectivity*. First, we tested whether PA and NA were related to this composite index. Second, we aimed to validate the specificity of the relationship between PA and SEN by testing whether there were significant relationships between PA and an affect-irrelevant intrinsic network, namely the default mode network (DMN), which is mainly associated with self-related cognitive activities and mind-wandering (Mason et al. 2007; Menon 2011). Third, we examined the strength and the directionality of the relationship between PA and the connectivity within the SEN, as well as the connectivity strengths between the SEN and other brain regions.

## Methods

### Participants

A total of 70 healthy adults participated in this study. All participants were right-handed, between 19 and 28 years old, and without a history of neurological or psychiatric disorders or contraindications for MRI scanning. They all completed a minimum of high school level of education. Written informed consents were obtained. This study was approved by the Human Research Ethics Committee of The University of Hong Kong. All experimental procedures followed the declaration of Helsinki.

Of the 70 enrolled participants, seven were excluded from the data analysis because of abnormally low non-verbal IQ score as assessed by the Test of Nonverbal Intelligence, Fourth Edition (TONI-4; Brown et al. 2010) (*n* = 1); abnormally high PA score that was three standard deviations (SDs) above the mean (*n* = 1); abnormal anxiety or depressive mood as measured by the Hospital Anxiety and Depression Scale (HADS; Snaith 2003) (*n* = 5). The final sample consisted of 63 participants.

Participants completed the questionnaires, cognitive assessment and MRI scanning in the same experimental session. They first completed the questionnaires (the Edinburgh Handedness Inventory-Short Form (EHI) (Veale 2014), HADS, Chinese Affect Scale (CAS; Hamid and Cheng 1996) and the cognitive assessment (TONI-4) outside the scanner, followed by the scanning in the MRI scanner.

### Affect measure

We used the Chinese Affect Scale (CAS; Hamid and Cheng 1996) to measure trait PA and NA. Participants were asked to indicate how they generally felt on a 5-point scale (from *very slightly or not at all* to *extremely*) so to provide scores of their affective disposition. The CAS was shown to be a valid measure of positive and negative affect for Chinese-speaking people with good internal consistency, test-retest reliability, and convergent and discriminant validity (Hamid and Cheng 1996).

### Imaging data acquisition and preprocessing

Brain images were acquired via a 3.0 Tesla Siemens Prisma scanner equipped with a 64-channel head coil at Peking University. Using a multiband echo-planar imaging (EPI) sequence (64 contiguous slices, 240 volumes, echo time (TE) = 30 ms, repetition time (TR) = 2000 ms, field of view (FOV) = 224 × 224 mm^2^, flip angle = 90°, and voxel size = 2 × 2 × 2.2 mm^3^) with a multiband factor of 2, the whole-brain rs-fMRI data were obtained. The CAIPIRINHA (Controlled Aliasing In Parallel Imaging Results IN Higher Acceleration) technique was applied with an acceleration factor of 2. Participants were instructed to keep their eyes open during the resting-state scanning, which lasted for 8 min. High-resolution anatomical T1-weighted images were collected using a three-dimensional magnetization-prepared rapid gradient echo (MP-RAGE) sequence (192 contiguous slices, TE = 2.98 ms, TR = 2530 ms, FOV = 224 × 256 mm^2^, flip angle = 7°, and voxel size = 0.5 × 0.5 × 1 mm).

The rs-fMRI data were preprocessed using Statistical Parametric Mapping (SPM12, Wellcome Department of Cognitive Neurology, London, UK) and the Data Processing Assistant for Resting-State fMRI (DPARSF, http://rfmri.org/DPARSF). The first ten volumes were discarded for magnetic field stabilization. The remaining data were corrected for the acquisition time delay between slices within a volume and further realigned to the first volume to correct for head motion. The corrected functional images were spatially normalized to the Montreal Neurological Institute (MNI) space using Diffeomorphic Anatomical Registration using Exponentiated Lie algebra (DARTEL) (Ashburner 2007) and were resampled to 3-mm isotropic voxels. The normalized functional images were further smoothed using a Gaussian kernel with full-width at the half maximum (FWHM) of 4 mm. The linear trend was removed for the time series of each voxel, and nuisance signals including the Friston’s 24 head motion parameters (Friston et al. 1996), white matter and cerebrospinal fluid signals (Yan et al. 2013) were regressed out. The Friston’s 24 head motion parameters were used here as this higher-order model was suggested to minimize the effects of head motion (Satterthwaite et al. 2013). Power frame displacement greater than 0.5 was deemed a “bad” time point, and the time points before and after that bad time point were scrubbed using each of the bad time points as a regressor (Power et al. 2012). Notably, we did not regress out global signal because this procedure remains controversial (Murphy and Fox 2017). Finally, the time courses were temporally band-pass filtered at 0.01–0.1 Hz to reduce the effect of low-frequency drifts and high-frequency physiological noise (Lowe et al. 1998).

### Resting-state functional connectivity (rs-FC) analysis

**Fig. 1:**
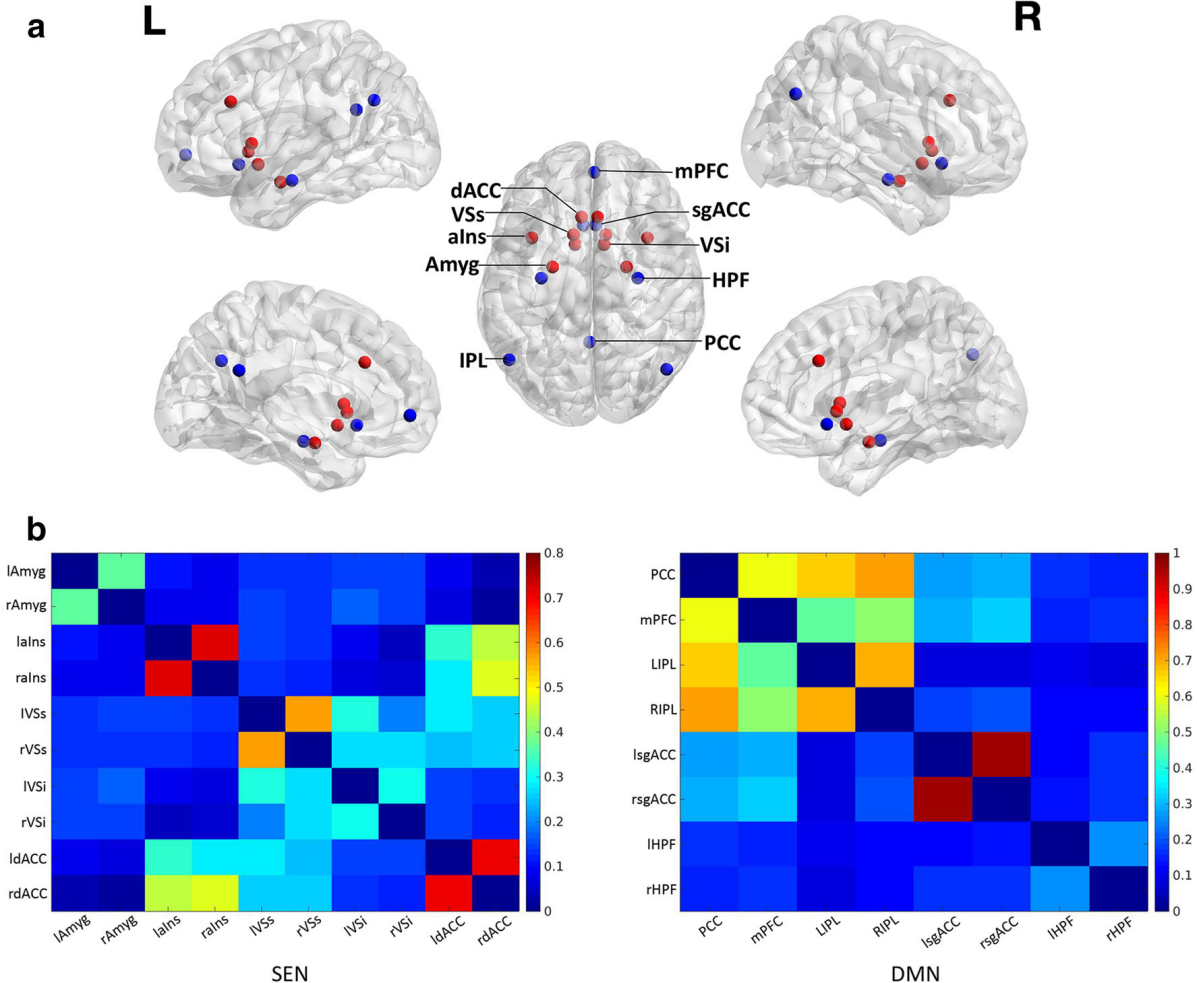
**a,** ROIs of the SEN (red) and the DMN (blue), and **b**, the correlation matrix depicting the pairwise correlations between all the ROIs of the SEN (left side) and the DMN (right side). **a**, either hemisphere for the bilateral nodes in the dorsal view was labelled. Amyg, amygdala; aIns, anterior insula; VSs, superior ventral striatum; VSi, inferior ventral striatum; dACC, dorsal anterior cingulate cortex; PCC, posterior cingulate cortex; mPFC, medial prefrontal cortex; IPL, left inferior parietal lobule; sgACC, subgenual anterior cingulate; HPF, hippocampal formation; L, left; R, right. **b**, the correlations were Fisher *z*-transformed. The color bar denoted Fisher *z*-transformed correlation coefficients

We adopted an ROI (region of interest)-based approach to analyze resting-state functional connectivity (rs-FC). We defined ROIs for the SEN and DMN as spherical volumes, each 5 mm in radius according to the conventional practice of defining ROIs in the literature (Calamante et al. 2012; Li et al. 2010). ROIs of the SEN were centered on MNI coordinates of ±23, −5, −19 for the bilateral amygdala (Amyg), on ±36, 13, 5 for the bilateral anterior insula (aIns), on ±10, 15, 0 for the bilateral superior ventral striatum (VSs), on ±9, 9, −8 for the bilateral inferior ventral striatum (VSi) (Bhaumik et al. 2017), and on ±5, 26, 31 for the bilateral dorsal anterior cingulate cortex (dACC) (Baur et al. 2013). We defined ROIs for the DMN with the posterior cingulate cortex (PCC) centered on 0, −52, 26, the medial prefrontal cortex (mPFC) centered on 3, 54, −2, the left inferior parietal lobule (LIPL) centered on −50, −63, 32, the right inferior parietal lobule (RIPL) centered on 48, −69, 35 (Di and Biswal 2014), the bilateral subgenual anterior cingulate (sgACC) centered on ±4, 21, −8, and the bilateral hippocampal formation (HPF) centered on ±30, −12, −18 (Bhaumik et al. 2017). We divided the ventral striatum into superior and inferior segments (VSs and VSi) referring to the ventral caudate and nucleus accumbens, respectively, as they have been shown to have distinct functional connections to other brain areas (Di Martino et al. 2008). The locations for each seed ROI of the SEN and the DMN are summarized in Fig. 1.

Next, averaged time courses for each ROI were extracted for each participant. Correlation coefficients were calculated between seed regions and transformed to *z* scores using Fisher transformation to get the FC values of each pair of ROIs. To examine the within-network connectivity in the DMN and the SEN, we averaged the FC values of each ROI pair within each network in the correlation matrix to get the composite metrics of “*DMN connectivity*” and “*SEN connectivity*”. Then we examined the relationship of PA with *SEN connectivity* and with *DMN connectivity* respectively. We performed the same analyses for NA. Then we conducted correlation analyses between PA/NA and the FC between each pair of distinct ROIs within the respective network if there was a significant relation with the composite *SEN/DMN connectivity*. Finally, to explore the connectivity profile related to the intrinsic network underpinning PA/NA from a whole-brain perspective, we first conducted voxel-wise analyses to get the FC maps of each ROI within the respective network with the whole brain, followed by multiple regression analyses to identify significant relationships between PA and the FC maps.

### Statistical analysis

All statistical analyses were performed using SPSS version 22 (IBM Corp.) and SPM 12. Gender differences of PA/NA were examined with an independent-samples *t* test, and Pearson correlation analyses were used to detect the relationships of PA/NA with age, *SEN connectivity*, *DMN connectivity*, and FC between ROIs of the intrinsic network. The threshold of statistical significance (*p*) was set at .05 for all analyses. For the relationship of PA with the FC between ROIs within the SEN, we conducted a multiple comparison correction (Bonferroni correction) within these six families: the FC between Amyg and aIns, the FC between Amyg and VS (including VSi and VSs), the FC between aIns and VS, the FC between dACC and Amyg, the FC between dACC and VS, and the FC between dACC and aIns. Multiple linear regressions were conducted to test the relationships of PA with the FC between each ROI of the SEN and the whole brain. Since separate multiple linear regression analyses were conducted for the 10 ROIs within the SEN, we applied the false discovery rate (FDR) correction at *p* < .005 (.05/10), and cluster size >10 (Lieberman and Cunningham 2009).

### Spectral dynamic causal modeling (spDCM) analysis

To estimate the causal interactions between regions in the SEN at rest whose FC showed significant correlations with PA, we performed spectral dynamic causal modeling (spDCM) for each individual using SPM12 (Friston et al. 2014). It was performed by (1) selecting ROIs within the SEN whose FC showed significant correlations with PA as volumes of interest (VOIs); (2) extracting the first eigenvariate of the time courses in the seed VOIs after removing the effects of head motion and signals from cerebrospinal fluid and white matter; (3) specifying and estimating all possible models; and (4) performing a family-level Bayesian model selection (BMS) analysis by grouping model subsets based on some common features of those models, with random effects model inference (Penny et al. 2010). We focused on exploring the directionality of the functional couplings between the VOIs for each VOI pair (i.e., bidirectional or unidirectional from one to another). The family with a posterior probability >95% was the winning family (Stephan et al. 2010).

## Results

### Demographic and behavioral characteristics of participants

Participants’ demographic and behavioral characteristics are presented in Table 1. PA showed positive correlations with age (*r* (61) = .34, *p* = .007) but did not differ between two genders (*t* (61) = 0.73, *p* = .47, so we did the following correlations with rs-FC controlling for age. In contrast, NA measures did not correlate with age (*r* (61) = −.17, *p* = .17) or differ between genders (*t* (61) = −0.45, *p* = .65). Further, there was no correlation between PA and NA (*r* (61) = −.048, *p* = .71).

**Table 1:** Demographic and behavioral characteristics of participants (*N* = 63)

Variables	Mean(SD)	Range
Age (years)	23.86 (2.29)	19–28
Gender	33 females	
Years of education	17.27 (2.05)	13–23
IQ (TONI-4) (*n* = 62)	102.63 (9.29)	86–122
HADS-Anxiety	4.56 (2.61)	0–10
HADS-Depression	3.71 (2.65)	0–9
CAS-Positive affect	33.71 (5.02)	21–48
CAS-Negative affect	19.67 (4.99)	10–31

TONI-4 is Test of Nonverbal Intelligence, Fourth Edition; HADS is the Hamilton Hospital Anxiety and Depression Scale; CAS is the Chinese Affect Scale. Notes: One datum on IQ measurement was missing because the measurement of TONI-4 of one participant was interrupted by technical problem. The participant had already reached the normal range of IQ when testing was interrupted

### Relationships of PA with rs-FC

The FC matrices depicting the pairwise correlations between all ROIs of the SEN and the DMN are shown in Fig. 1. The partial correlation analysis between PA and *SEN connectivity* controlled for age indicated a significant negative correlation (*r* (60) = −.25, *p* = .05). As expected, PA did not correlate with *DMN connectivity* (*r* (60) = −.16, *p* = .22). For NA, Pearson correlation analyses showed neither correlation with *SEN connectivity* (*r* (61) = .05, *p* = .68) nor with *DMN connectivity* (*r* (61) = .12, *p* = .36).

**Fig. 2:**
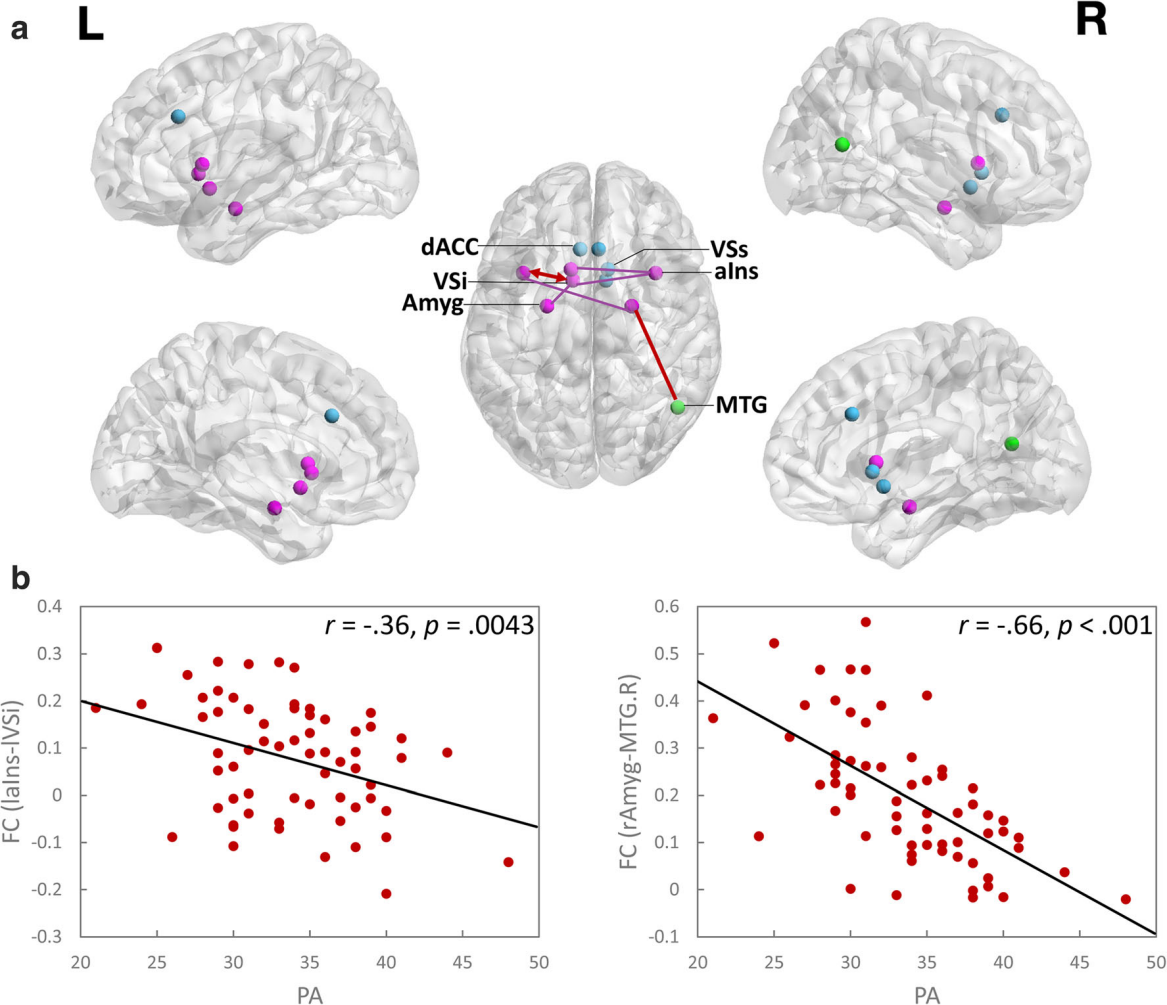
**a**, the SEN related FC which were negatively correlated with PA, and **b**, scatter plots of the relationships between PA and the FC between laIns and lVSi (left side), and the FC between rAmyg and MTG.R (right side). **a**, either hemisphere for the bilateral nodes in the dorsal view was labelled. Those within-SEN connectivities which showed correlations with PA were shown in purple (related nodes were also in purple, other nodes in SEN were in blue). The FC between the left aIns and left VSi within SEN, and FC between right Amyg and right MTG (in green) survived the multiple corrections and were shown in red. The bidirectional arrow between the left aIns and left VSi showed the bidirectional effective connectivity between them. Amyg, amygdala; aIns, anterior insula; VSs, superior ventral striatum; VSi, inferior ventral striatum; dACC, dorsal anterior cingulate cortex; MTG, middle temporal gyrus. L, left; R, right. **b**, FC, functional connectivity; laIns, left anterior insula; lVSi, left inferior ventral striatum; rAmyg, right amygdala; MTG.R, right middle temporal gyrus. The partial correlation between PA and the FC between laIns and lVSi controlled for age is *r* (60) = −.36 (*p* = .0043). The partial correlation between PA and the FC between laIns and lVSi controlled for age is *r* (60) = −.66 (*p* < .001)

The partial correlation analyses conducted between PA and the FC between pairs of distinct ROIs within the SEN showed that PA was negatively correlated with the FC between the left aIns and the left VSi, after the Bonferroni correction at *p* ≤ .05 level (Table 2, Fig. 2).

**Table 2:** Significant correlations of PA with FC between each pair of ROIs of the SEN

rs-FC		PA	
		*r*	*p*
Amyg - aIns	rAmyg – laIns	−.30	.016
Amyg – VS	lAmyg – lVSi	−.28	.028
aIns – VS	laIns – lVSi raIns – lVSs raIns – lVSi	−.36 −.32 −.31	.0043* .012 .016
dACC - Amyg	None	–	–
dACC - aIns	None	–	–
dACC – VS	None	–	–

*PA* positive affect, *rs-FC* resting-state functional connectivity, *Amyg* amygdala, *aIns* anterior insula, *VS* ventral striatum, *dACC* dorsal anterior cingulate cortex, *rAmyg* right amygdala, *lAmyg* left amygdala, *lVSi* left inferior ventral striatum, *laIns* left anterior insula, *raIns* right anterior insula, *lVSs* left superior ventral striatum. The correlations shown here were all controlled for age. The statistical significance was set at *p* ≤ .05

*Significant after a Bonferroni correction at *p* ≤ .05

Multiple linear regressions for each of the FC maps of seed ROIs of the SEN with PA as the predictor and with age as the covariate showed that only the FC between right amygdala (rAmyg) and a cluster of the right middle temporal gyrus (MTG.R) (peak intensity voxel coordinate: *x* = 48, *y* = −60, *z* = 15, peak intensity: −6.11, cluster size: 20) was correlated with PA (*r* (60) = −.66, *p* < .001) at FDR correction level of *p* < .005, and a cluster size > 10 (Fig. 2). However, no relations were found for PA and FC between rAmyg and other brain regions, nor for PA and FC between other seed ROIs of the SEN and the brain.

### Spectral dynamic causal modeling (spDCM) analysis

**Fig. 3:**
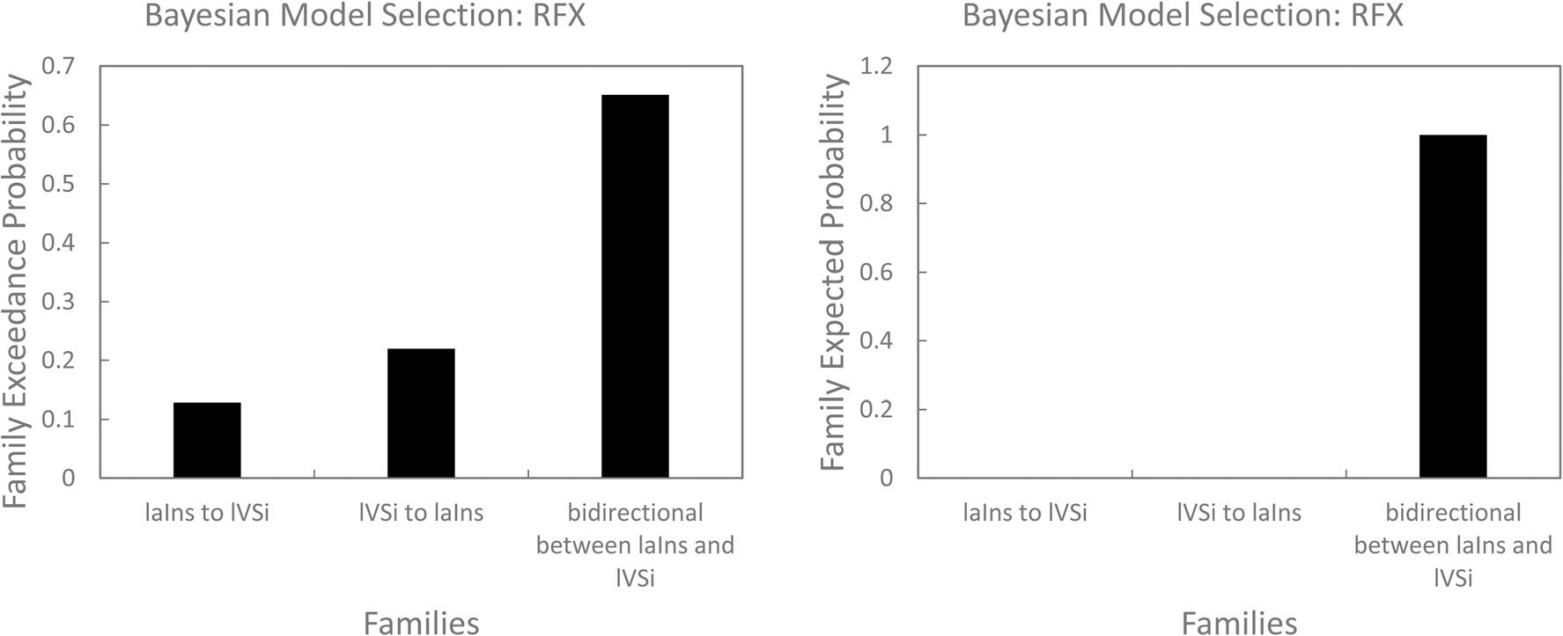
Results of a Bayesian Model Selection for the data showing that the third family, defined by bidirectional connections between the left anterior insula (laIns) and the left inferior ventral striatum (lVSi), fits the data best compared to the other two families representing unidirectional connections from laIns to lVSi and unidirectional connections from lVSi to laIns

As the FC between laIns and lVSi within the SEN, and the FC between rAmyg and MTG.R showed significantly negative correlations with PA, we selected laIns, lVSi, and rAmyg within the SEN as VOIs. We mainly focused on exploring the directionality of the FC between laIns and lVSi, two regions that showed significant within-SEN connectivity. Therefore, the models were grouped into three families (i.e., bidirectional, laIns to lVSi, and lVSi to laIns). A total of 3 × 2^4^ = 48 models (see Supplemental Figure) were estimated for each individual, with 16 models in each family. The result showed the winning family was the bidirectional connectivity between laIns and lVSi (probability = 1) (Fig. 3).

## Discussion

The current study investigated how positive and negative affect were associated with the intrinsic connectivity of brain regions within the SEN and DMN. The key findings of this study were PA’s negative associations with SEN connectivity, particularly between the left anterior insula and the left nucleus accumbens. However, there were no statistically significant relationships between PA and the DMN connectivity or between NA and the SEN/DMN connectivity. For connections between the SEN and the rest of the brain, we observed that positive affect was negatively related to the connectivity between the right amygdala and the right middle temporal gyrus.

One important role of the SEN is to detect salient events that are pleasurable, emotionally engaging, or self-relevant (Menon 2015). Individuals with stronger trait PA are likely to be more ready in detecting salient events. NA is dimensionally different from PA: it reflects how easily an individual is involved in aversive mood states and low NA suggests a state of calmness and serenity (Watson et al. 1988). In our study, we observed a negative association between PA and connectivity within SEN. This finding was consistent with Rohr et al.’s study (2013) where they observed a large network negatively correlated with PA. The negative correlation suggests that individuals with a higher PA trait may consume fewer connectivity resources to detect salient events. Moreover, consistent with our hypothesis, PA was not associated with the DMN connectivity as the DMN is mainly implicated in cognitive processing, suggesting that PA’s association with the SEN was unique.

The anterior insula subserves the detection and selection of salient stimuli and plays key roles in emotional awareness, interoception, and the generation of subjective feelings (Craig 2009), while the nucleus accumbens is critically associated with reward and motivation. The connectivity between anterior insula and the inferior ventral striatum might play roles in processing appetitive stimuli and generating feelings of happiness, so it was unsurprisingly, found to relate to PA. The bidirectional connection between the anterior insula and the nucleus accumbens found suggested that the two regions interacted with each other rather than unidirectionally from one to another.

It is worth noting the strong relationship between PA and the connectivity between the right amygdala and the right middle temporal gyrus. Previous reports have suggested that the middle temporal gyrus was involved in emotional, social cognitive, and language and semantic processes. It also plays a significant role in multi-model sensory integration (Cabeza and Nyberg 2000; Lu et al. 2014; Mesulam 1998; Whitney et al. 2010). Furthermore, Lu et al. (2014) observed a negative relationship between extraversion and gray matter volume of bilateral amygdalae as well as the right middle temporal gyrus. Following this line of thought, our findings further highlight the role of the amygdala and middle temporal gyrus in emotion processing, specifically, the role of the FC between the amygdala and middle temporal gyrus in PA.

There were a few limitations of this study. First, we employed a cross-sectional design that could not infer the causality for the observed significant relationships. Second, we measured PA at one time point in this study. We fully appreciate that PA may change over time and that multiple measurements of PA could better capture the neural connectivity pattern underpinning PA. Lastly, the age range of our sample was small, hence hindering our ability to generalize our findings to other age cohorts.

## Conclusions

Our study supports that affect-based modulations of connectivity were specific to PA and the SEN network. The neural connectivity underpinning PA highlights the critical role of the SEN in promoting emotional health and lays the groundwork for future studies on modeling SEN connectivity to predict mental well-being.

## Electronic supplementary material

ESM 1 Supplemental Figure All the possible 48 models in the spectral dynamic causal modeling (spDCM) analysis. Models (1) – (16), the direction between laIns and lVSi is from laIns to lVSi; Models (17) – (32), the direction between laIns and lVSi is from lVSi to laIns; Models (33) – (48), the direction between laIns and lVSi is bidirectional. laIns, left anterior insula; lVSi, left inferior ventral striatum; rAmyg, right amygdala. (PNG 713 kb)

High resolution image (TIF 56755 kb)
