# Supplementary figures and images for: Positive affect is inversely related to the salience and emotion network’s connectivity
Source: Brain Imaging Behav. 2020 Oct 8;15(4):2031–9. doi: 10.1007/s11682-020-00397-1 (PMC8413151; doi:10.1007/s11682-020-00397-1)

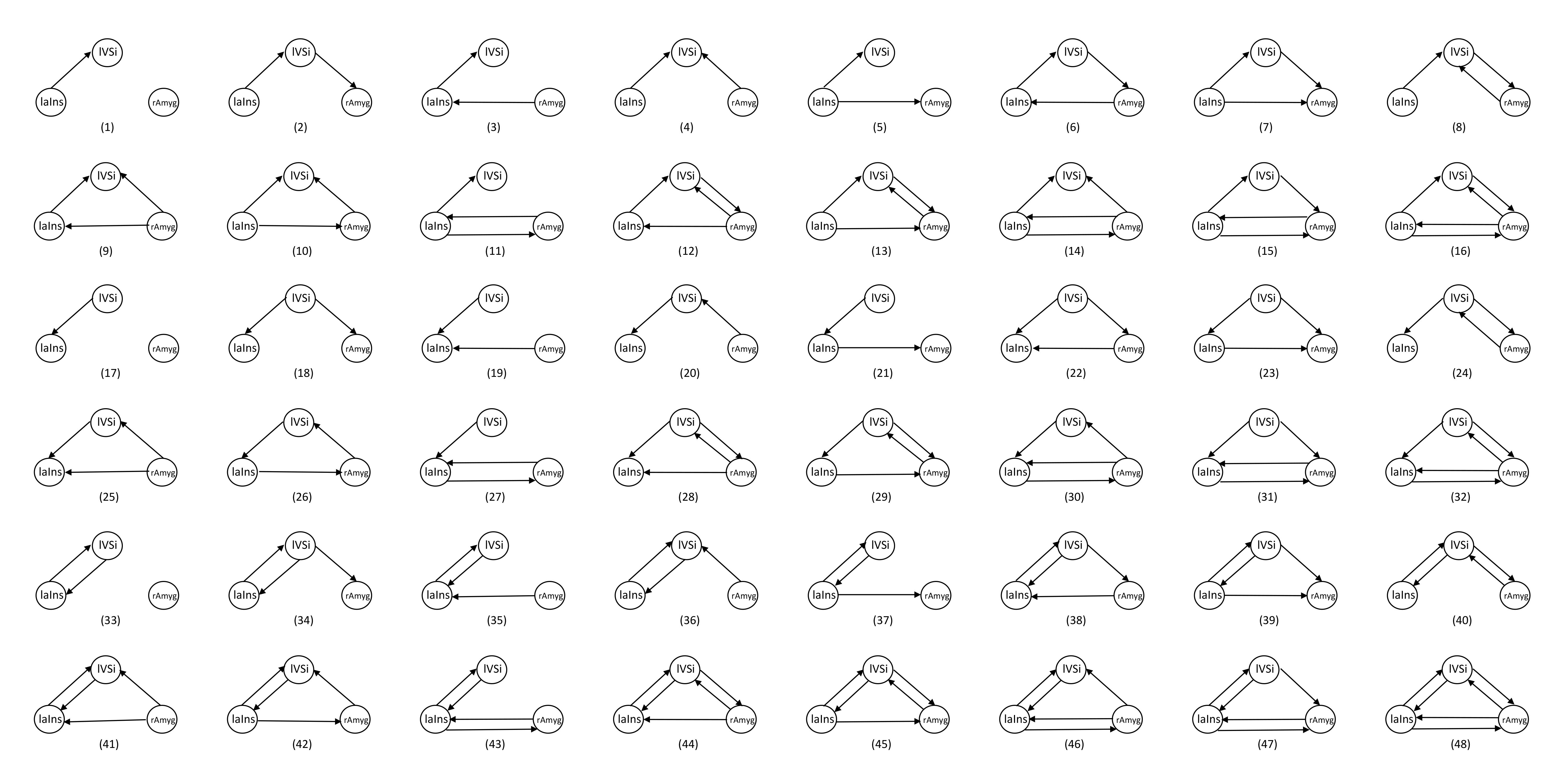

Supplement: Supplementary file 1 — Supplemental Figure All the possible 48 models in the spectral dynamic causal modeling (spDCM) analysis. Models (1) – (16), the direction between laIns and lVSi is from laIns to lVSi; Models (17) – (32), the direction between laIns and lVSi is from lVSi to laIns; Models (33) – (48), the direction between laIns and lVSi is bidirectional. laIns, left anterior insula; lVSi, left inferior ventral striatum; rAmyg, right amygdala. (PNG 713 kb) [file 11682_2020_397_Fig4_ESM.png]

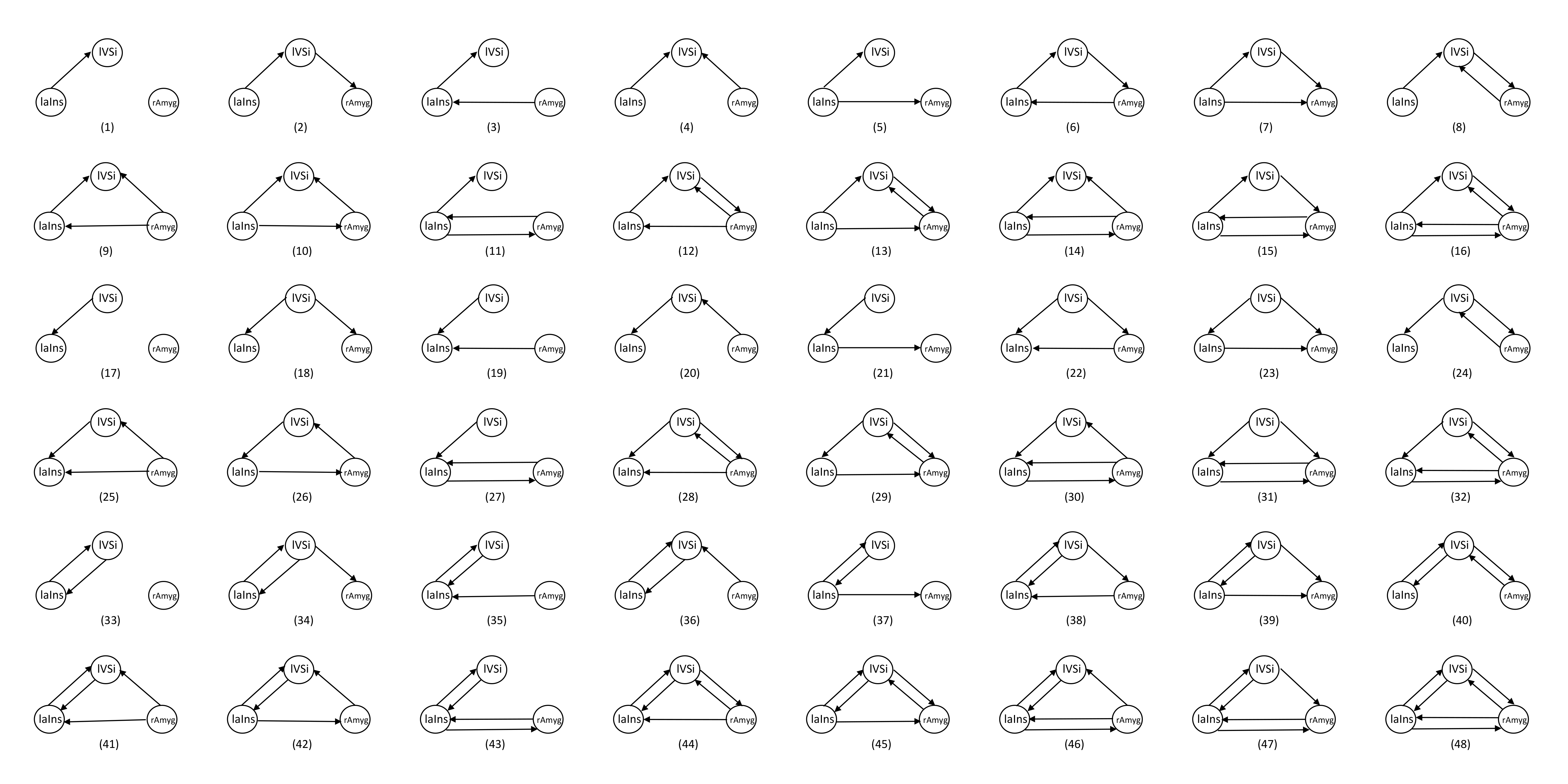

Supplement: Supplementary file 2 — High resolution image (TIF 56755 kb) [file 11682_2020_397_MOESM1_ESM.tif]
